# Hyperspectral imaging of 4T1 mammary carcinomas grown in dorsal skinfold window chambers: spectral renormalization and fluorescence modeling

**DOI:** 10.1117/1.JBO.29.9.093504

**Published:** 2024-07-22

**Authors:** Tadej Tomanic, Tim Bozic, Bostjan Markelc, Jost Stergar, Gregor Sersa, Matija Milanic

**Affiliations:** a University of Ljubljana, Faculty of Mathematics and Physics, Ljubljana, Slovenia; b Institute of Oncology Ljubljana, Department of Experimental Oncology, Ljubljana, Slovenia; c Jozef Stefan Institute, Ljubljana, Slovenia; d University of Ljubljana, Faculty of Health Sciences, Ljubljana, Slovenia

**Keywords:** hyperspectral imaging, tumors, dorsal skinfold window chambers, renormalization, enhanced green fluorescent protein, tissue model

## Abstract

**Significance:**

Hyperspectral imaging (HSI) of murine tumor models grown in dorsal skinfold window chambers (DSWCs) offers invaluable insight into the tumor microenvironment. However, light loss in a glass coverslip is often overlooked, and particular tissue characteristics are improperly modeled, leading to errors in tissue properties extracted from hyperspectral images.

**Aim:**

We highlight the significance of spectral renormalization in HSI of DSWC models and demonstrate the benefit of incorporating enhanced green fluorescent protein (EGFP) excitation and emission in the skin tissue model for tumors expressing genes to produce EGFP.

**Approach:**

We employed an HSI system for intravital imaging of mice with 4T1 mammary carcinoma in a DSWC over 14 days. We performed spectral renormalization of hyperspectral images based on the measured reflectance spectra of glass coverslips and utilized an inverse adding–doubling (IAD) algorithm with a two-layer murine skin model, to extract tissue parameters, such as total hemoglobin concentration and tissue oxygenation (${\mathrm{StO}}_{2}$). The model was upgraded to consider EGFP fluorescence excitation and emission. Moreover, we conducted additional experiments involving tissue phantoms, human forearm skin imaging, and numerical simulations.

**Results:**

Hyperspectral image renormalization and the addition of EGFP fluorescence in the murine skin model reduced the mean absolute percentage errors (MAPEs) of fitted and measured spectra by up to 10% in tissue phantoms, 0.55% to 1.5% in the human forearm experiment and numerical simulations, and up to 0.7% in 4T1 tumors. Similarly, the MAPEs for tissue parameters extracted by IAD were reduced by up to 3% in human forearms and numerical simulations. For some parameters, statistically significant differences (p<0.05) were observed in 4T1 tumors. Ultimately, we have shown that fluorescence emission could be helpful for 4T1 tumor segmentation.

**Conclusions:**

The results contribute to improving intravital monitoring of DWSC models using HSI and pave the way for more accurate and precise quantitative imaging.

## 1 Introduction

Tumors and their microenvironment must be adequately understood for effective diagnosis and treatment strategies. The hallmarks of cancer are sustained proliferation, evasion of growth suppressors, resistance to cell death, activation of invasion and metastasis, inflammation, and metabolic specificity.^1^^,^^2^ Tumors are also known to induce angiogenesis, forming irregular and immature blood vessels that could lead to hyperpermeability, hypoxia, and reduced blood flow within the tumor.^3^^–^^6^ The complex vasculature poses a significant challenge to various cancer treatments by creating barriers to drug delivery, fostering aggressive tumor behavior, and decreasing treatment efficacy, particularly in radiotherapy, chemotherapy, and immunotherapy.^7^^–^^9^ Thus, understanding these dynamics is crucial in mitigating therapy side effects and improving treatment outcomes.

Dorsal skinfold window chambers (DSWCs) have emerged as indispensable tools in experimental tumor imaging due to their unique capacity to enable real-time, high-resolution imaging of tumor progression and response to treatment. DSWCs enable intravital visualization of tumor microvasculature and dynamics, offering insights into vascular morphology, angiogenesis, and overall tumor growth. The ability to longitudinally track these changes aids in assessing the efficacy of therapeutic approaches and understanding the underlying mechanisms involved in tumor development.^10^^–^^12^

DSWCs are usually paired with diagnostic optical imaging methods that detect reflected, transmitted, or fluorescent light propagating from biological tissues, such as tumors, toward the light detector. The light interacting with the tissues carries essential information about the tissue—optical properties that can be determined include tissue absorption (e.g., blood and melanin content) and scattering (e.g., morphology).^13^^,^^14^

Biomedical hyperspectral imaging (HSI) represents a promising non-invasive and contactless optical technique combining imaging and spectroscopy, capturing both spatial and spectral data in a hyperspectral image (hypercube).^15^ Thus, the resulting hyperspectral images contain spectral information within each image pixel. Typically, HSI operates in the visible and near-infrared spectral bands.^16^ The imaging system can be configured to work in various modes, capturing reflected, transmitted, or fluorescent light from the investigated biological tissues.^15^

The hyperspectral image analysis provides valuable diagnostic insights into tissue physiology, pathology, morphology, and structure. By utilizing the extracted tissue properties, HSI proves helpful in distinguishing various diseases, such as cancers, heart and circulatory pathologies, retinal diseases, gastrointestinal diseases, and skin diseases.^15^^,^^17^

Several studies have used HSI to image subcutaneously grown murine tumor models implanted in DSWCs. The majority of them studied tumor hemodynamics, especially tissue oxygenation (${\mathrm{StO}}_{2}$), and hypoxia.^18^^–^^26^ Certain studies utilized various tumor treatment approaches and observed their effect using HSI. Lee et al.^22^ treated tumors with vascular targeting agents. Choe et al.^24^ showcased the enhancement effect of photosensitization on the delivery of a model therapeutic encapsulated in murine sickle red blood cells. McKee et al.^27^ treated tumors with the epidermal growth factor receptor-targeted high-density lipoprotein nanoparticles. Most studies employing HSI and DSWCs performed longitudinal studies spanning several days,^18^^,^^22^^–^^25^^,^^27^ whereas some acquired hyperspectral images at a single time point^19^ or monitored dynamics in the span of several minutes^26^ or hours.^20^

All studies mentioned above determined specific tissue properties (e.g., ${\mathrm{StO}}_{2}$ or hypoxia extent) from hyperspectral images based on spectral features of investigated tissues. However, only selected studies used tumor cells expressing enhanced green fluorescent protein (EGFP) to localize the tumor and hypoxic regions.^18^^,^^19^^,^^27^ Moreover, the HSI of tumors implanted in DSWCs requires light to pass through a glass coverslip. In reflectance mode,^15^ the light traverses the coverslip during illumination (incident light) and after interacting with the tissue (outgoing light). Due to the imperfect transmission of the glass coverslip, the reflected light exhibits reduced intensity, resulting in an underestimation of tissue reflectance. Consequently, this discrepancy impacts the derived values of tissue properties extracted from hyperspectral images that describe investigated tissues (e.g., tumors).

This study used a custom-built HSI system integrated with three-dimensional (3D) optical profilometry (OP) for intravital monitoring of mice with 4T1 mammary carcinomas (from now on called 4T1 tumors) implanted in the DSWC in a span of 14 days following tumor cell injection. The main goal of this work is to showcase the importance of renormalizing the hyperspectral images to compensate for the signal loss due to incoming and backscattered light traversing the glass coverslip in a DSWC to obtain the correct values of tissue properties. Because the 4T1 tumor cells expressed a gene to produce EGFP, our second goal is to show that incorporating EGFP fluorescence excitation and emission in the skin tissue model improves the performance of the inverse adding–doubling (IAD) algorithm for the extraction of 4T1 tumor properties from hyperspectral images. Finally, our third goal is to show that EGFP fluorescence emission could be helpful for tumor localization and segmentation of 4T1 tumors expressing genes to produce EGFP. The proposed hyperspectral image renormalization process paves the way for more accurate and precise quantitative imaging by considering and compensating for errors inherent in experimental procedures. In addition, EGFP fluorescence modeling could enable the automatic segmentation of tumors expressing EGFP, thereby facilitating image processing and analysis. We support our findings with additional experiments involving HSI of tissue-mimicking phantoms and a human forearm skin and numerical simulations. Ultimately, we leverage spectral renormalization to evaluate changes in tissue properties of 4T1 tumors during the course of the experiment and showcase that HSI could help detect tumors at early stages.

## 2 Methods

### 2.1 Animal Handling

Animal experiments were conducted at the Department of Experimental Oncology, Institute of Oncology Ljubljana. This study used seven female inbred Balb/c (BALB/cAnNCrl, Charles River Laboratories, Wilmington, Massachusetts, United States) mice aged 6 to 8 weeks. The mice weighed between 18 and 20 g at the beginning of the experiments. During the experiments, they were housed in a specific pathogen-free environment maintained at 20°C to 24°C temperature with a 12-h light–dark cycle and 55%±10% relative humidity. Food and water were available *ad libitum*.

Twenty-four hours before the start of the experiment, DSWCs were surgically implanted on the backs of the mice, as described previously.^28^ Tumors were grown within DSWCs after subcutaneous injection of $3\times 10^{5}$ 4T1-EGFP mammary carcinoma cells in 100  μl of 0.9% NaCl saline. When the tumors reached 4 mm in diameter, a gene electrotransfer procedure was performed, as described previously.^29^ To follow systemic toxicity, the mice’s body weight was measured, and behavior was assessed using the mouse grimace scale.^30^

Approval of all ethical and experimental procedures and protocols in animals was granted by the Ministry of Agriculture, Forestry and Food of the Republic of Slovenia (permission no. U34401-3/2022/11). The experimental procedures complied with the European Union (EU) directive (2010/63/EU) and Animal Research: Reporting of *In Vivo* Experiments guidelines for animal experiments.

The sample size of seven mice in this study was carefully chosen, considering the severity of the DSWC mouse model procedure. By minimizing the number of subjects, we adhered to the 3R reduction principle.

### 2.2 Imaging Protocol

**Fig. 1:**
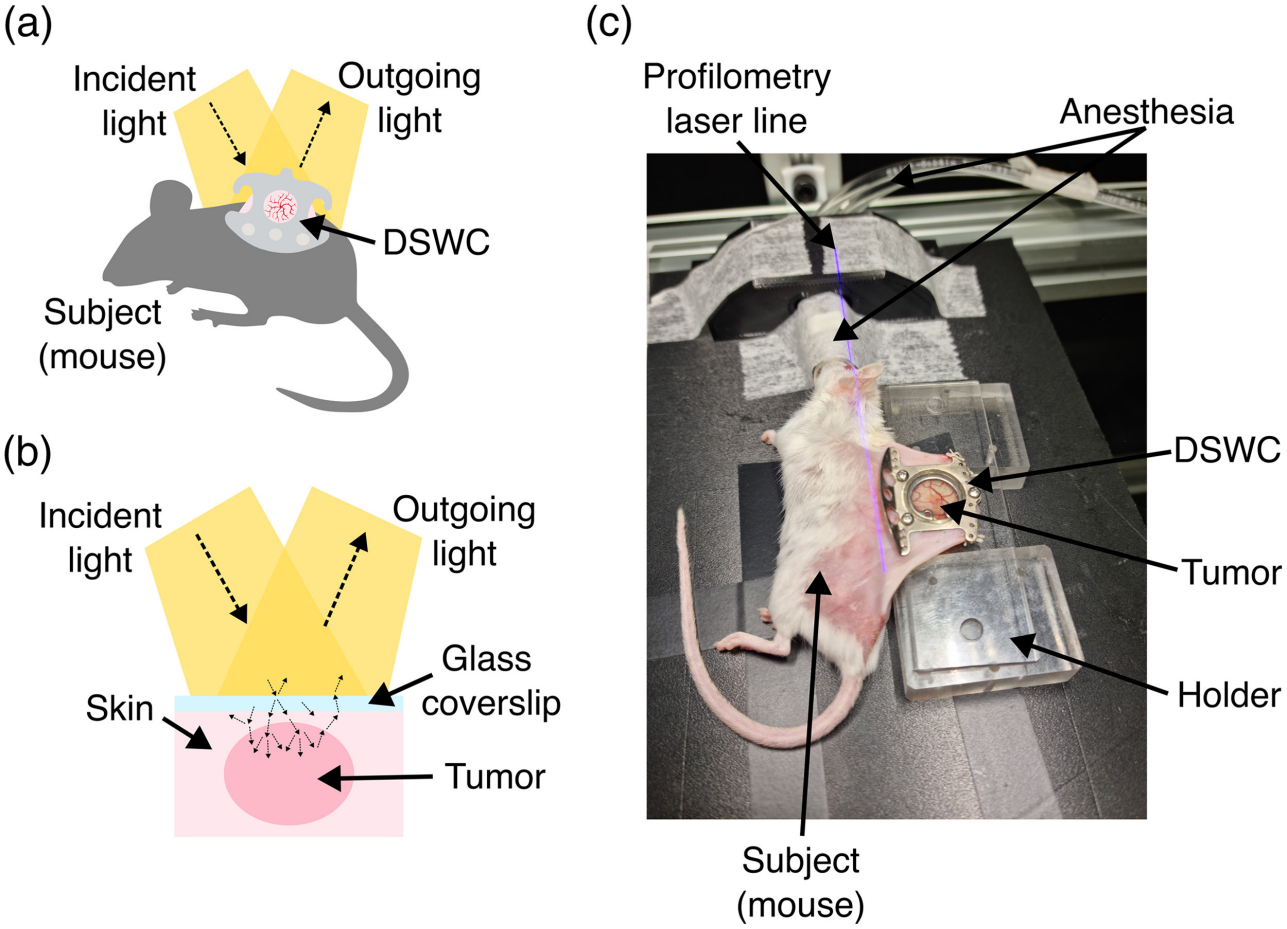
(a) Schematic representation of a subject (mouse) with a 4T1 tumor growing in a DSWC. (b) Cross-sectional view of a tumor growing in a DSWC. The light passes through the glass coverslip and interacts with the tissue (skin and tumor). Outgoing (reflected, remitted, and fluorescent) light is captured with the imaging system. (c) Animal experiment setup. The mouse is anesthetized during imaging, and the DSWC is fixed with a holder.

Intravital imaging of mice was performed using the integrated HSI and OP system for a period of 14 days, starting on the day of tumor cell injection (day 0), as seen in Fig. 1. Imaging was performed on days 0, 3 to 7, and 10 to 14. During image acquisition, mice were under 2% (v/v) of isoflurane anesthesia (isoflurane, Piramal Healthcare UK Limited, Northumberland, United Kingdom), and the DSWCs were fixed with a holder to reduce respiratory motion artifacts [see Fig. 1(c)].

### 2.3 Imaging System

The HSI system employed in this study is a custom-built integrated imaging system that combines a line-scanning HSI module with a 3D OP module. The system design and validation were thoroughly described by Stergar et al.^31^ Briefly, the HSI module spans the spectral range of 400 to 1000 nm with a spectral resolution of 2.9 nm and a spatial resolution of 100  μm in both the X and Y directions. The OP module is based on the laser triangulation method with a 405-nm line laser and achieves an accuracy of 100, 100, and 50  μm in the X, Y, and Z directions, respectively. The exposure time for a single line acquisition was set to 250 ms.

The integration of the HSI and OP modules enables the acquisition of the 3D surface shape of the imaged sample and allows for the application of curvature and height corrections to the hyperspectral images, as previously reported.^32^ These corrections address signal loss in hyperspectral images due to high surface inclination angles and large distances, facilitating reliable image processing and analysis.^33^ Multiple checkerboard measurements were conducted at various heights to ensure proper alignment of the two modules, resulting in a total image misalignment of less than 100  μm.^33^

### 2.4 Image Processing

Raw hyperspectral images are first normalized to obtain reflectance values:^15^

$$I_{\mathrm{ref}}=\frac{I_{\text{raw}}-I_{\text{dark}}}{I_{\text{white}}-I_{\text{dark}}}.$$ (1)

Here, $I_{\mathrm{ref}}$ is the sample reflectance, $I_{\text{raw}}$ is the raw sample intensity, $I_{\text{dark}}$ is the dark current intensity, and $I_{\text{white}}$ is the white standard intensity of light.

Then, curvature and height corrections were applied as part of our standard hyperspectral image preprocessing pipeline to compensate for the signal loss, as presented by Rogelj et al.^32^^,^^33^ Height correction mainly compensated for the tilted glass coverslips that were not completely parallel with the focal plane of the HSI system during imaging to reduce the specular reflection. By contrast, curvature correction was negligible because glass coverslip curvature was lower than 2 deg, and the cosine of the angle used for Lambert correction was close to 1. Furthermore, we performed a 2× spatial binning and 5× spectral reduction to speed up image analysis. The resulting processed hyperspectral image was a data cube with 612×300×61  pixels, of which the 61 spectral points are in the 450 to 750 nm range with a step of 5 nm.

**Fig. 2:**
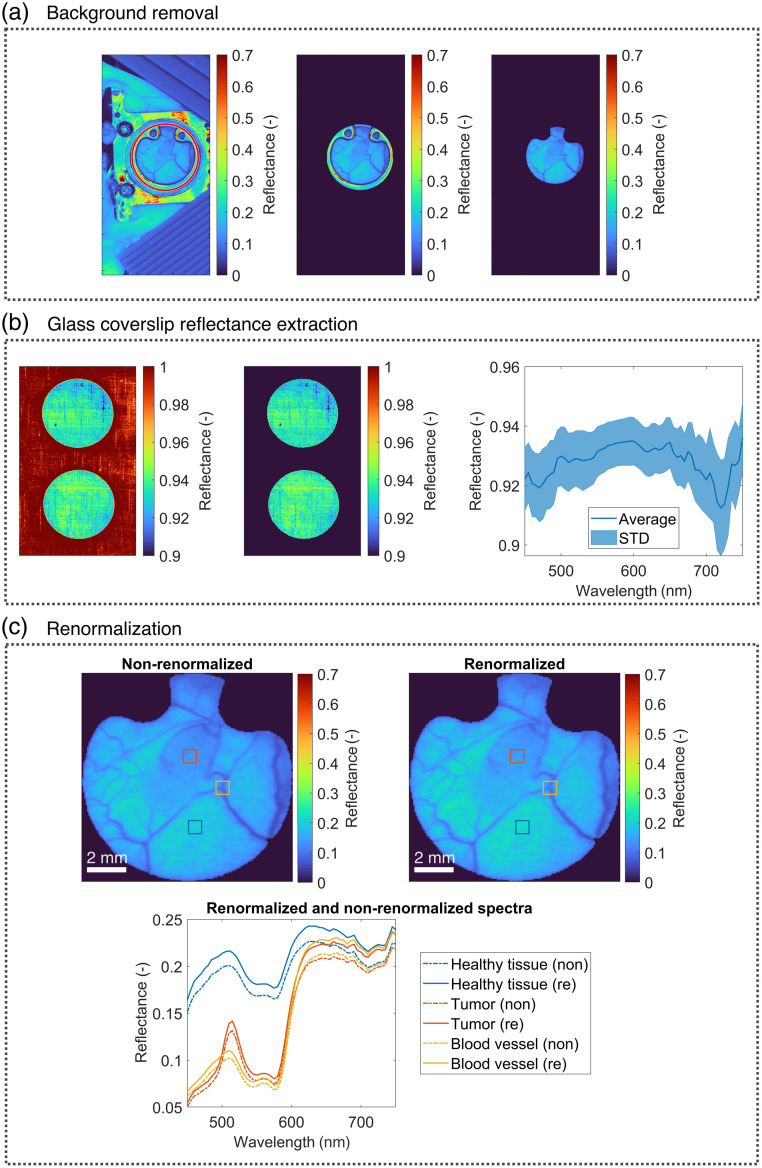
(a) Background removal from hyperspectral images (595-nm spectral band) by detecting DSWCs as circles (left, encircled in red), obtaining a binary mask, and masking out the area outside DSWCs (middle). Using a flood-fill algorithm, the DSWC ring was also removed (right). (b) Background removal from glass coverslip images (left, middle) to obtain the average reflectance properties of the white standard with the coverslips (right) and the corresponding standard deviations (shaded area). (c) Non-renormalized (top left) and renormalized (top right) 595-nm spectral band and the corresponding reflectance skin spectra (bottom) for various tissues. The plotted spectra are taken from the square regions of interest on the spectral band images.

The background (i.e., tissues and other structures outside the DSWCs) was removed from hyperspectral images by detecting the DSWCs based on their circular shape by first using the *imfindcircles* function in MATLAB R2022b (Mathworks, Natick, Massachusetts, United States) and then applying a flood-fill algorithm to remove the ring, as seen in Fig. 2(a). Because the skin within each DSWC was covered with a 12-mm (in diameter) glass coverslip (GL100, APJ Trading Co., Ventura, California, United States), we extracted the reflectance properties of two separate glass coverslips placed on the white reflectance standard (Spectralon, Labsphere Inc., North Sutton, New Hampshire, United States), as seen in Fig. 2(b). The average reflectance of the white standard with GL100 glass coverslips in the spectral range of 450 to 750 nm used for renormalization is shown in Fig. 2(b) (right) with the accompanying standard deviation values (shaded area). Given the homogeneous nature of the glass coverslips, with standard deviations within 1% of the average value, and the fact that the values were derived from over 50,000 spectra, we can reasonably assume the consistency of the spectral properties of glass coverslips. Although only two GL100 coverslips were tested (N=2), this sample size is deemed sufficient because the material is uniform. The homogeneity within individual coverslips suggests that the variability between different coverslips of the same kind should also be minimal. We could not obtain the actual transmittance or reflectance values of the glass coverslips from the vendor, but given our values, the transmittance is higher than 90%, a typical value for glass. Finally, we renormalized the hyperspectral images to compensate for the signal loss due to incoming and backscattered light passing through the coverslip. Hyperspectral images are renormalized as

$$I_{\mathrm{re}}=\frac{I_{\mathrm{ref}}}{I_{\text{coverslip}}},$$ (2)

where $I_{\mathrm{re}}$ is the renormalized sample reflectance, $I_{\mathrm{ref}}$ is the sample reflectance obtained from Eq. (1), and $I_{\text{coverslip}}$ is the average reflectance of the white standard with the coverslips, as seen in Fig. 2(b) (right). Examples of a 595-nm spectral band [Fig. 2(c), top] and three reflectance skin spectra before and after renormalization [Fig. 2(c), bottom] are also shown. The same reflectance spectrum shown in Fig. 2(b) (right) was used for all tissue types as the light loss was consistent across the entire area covered by the glass coverslip.

### 2.5 IAD Algorithm

We implemented the IAD algorithm to extract information about tissues from hyperspectral images. The IAD algorithm was enhanced using a graphics processing unit (GPU) to allow for fast and accurate light propagation simulation in layered turbid media.^34^ The accuracy and reliability of IAD for hyperspectral images were thoroughly tested and previously documented, along with specific details regarding the implementation of the algorithm and tissue modeling.^35^ In summary, a two-layer model of murine skin, consisting of an upper layer (epidermis) and a lower layer (dermis), was employed.^35^ Our original two-layer murine skin model employed 11 tissue parameters to characterize tissue physiology (e.g., melanin volume fraction, deoxy- and oxyhemoglobin volume fractions, molar concentration of bilirubin, and reduced and oxidized cytochrome C oxidase), morphology (e.g., scattering coefficient, scattering power, and the fraction of Rayleigh scattered light), and thicknesses of the epidermis and dermis.^35^^,^^36^

The details about epidermis and dermis absorption and tissue scattering were previously reported.^35^^,^^36^ Importantly, the dermis absorption is calculated as^35^^,^^36^

$$\mu_{a,d}=f_{\mathrm{Hb}}\mu_{a,\mathrm{Hb}}+f_{{\mathrm{HbO}}_{2}}\mu_{a,{\mathrm{HbO}}_{2}}+f_{\text{brub}}\mu_{a,\text{brub}}+f_{\mathrm{CO}}\mu_{a,\mathrm{CO}}+f_{{\mathrm{COO}}_{2}}\mu_{a,{\mathrm{COO}}_{2}}+\mu_{a,\text{base}},$$ (3)

where $f_{\mathrm{Hb}}$ and $f_{{\mathrm{HbO}}_{2}}$ are volume fractions of deoxy- and oxyhemoglobin, respectively; $\mu_{a,\mathrm{Hb}}$ and $\mu_{a,{\mathrm{HbO}}_{2}}$ are the corresponding absorption coefficients; and $f_{\text{brub}}$ and $\mu_{a,\text{brub}}$ are the molar concentration and absorption coefficient of bilirubin, respectively. Moreover, $f_{\mathrm{CO}}$ and $f_{{\mathrm{COO}}_{2}}$ are the molar concentrations of reduced and oxidized cytochrome C oxidase, respectively, and $\mu_{a,\mathrm{CO}}$ and $\mu_{a,{\mathrm{COO}}_{2}}$ are the associated absorption coefficients. Finally, $\mu_{a,\text{base}}$ is the baseline absorption of bloodless skin.

Because the tumor cells expressed a gene to produce EGFP, the dermis absorption coefficient is adapted to consider EGFP excitation (absorption) and emission:

$$\mu_{a,d}=f_{\mathrm{Hb}}\mu_{a,\mathrm{Hb}}+f_{{\mathrm{HbO}}_{2}}\mu_{a,{\mathrm{HbO}}_{2}}+f_{\mathrm{brub}}\mu_{a,\text{brub}}+f_{\mathrm{CO}}\mu_{a,\mathrm{CO}}+f_{{\mathrm{COO}}_{2}}\mu_{a,{\mathrm{COO}}_{2}}\;+f_{{\mathrm{EGFP}}_{\mathrm{ex}}}\mu_{{a,\mathrm{EGFP}}_{\mathrm{ex}}}-f_{{\mathrm{EGFP}}_{\mathrm{em}}}\mu_{{a,\mathrm{EGFP}}_{\mathrm{em}}}+\mu_{a,\text{base}}.$$ (4)

In Eq. (4), $f_{{\mathrm{EGFP}}_{\mathrm{ex}}}$ and $f_{{\mathrm{EGFP}}_{\mathrm{em}}}$ are the parameters describing the intensity of EGFP excitation and emission, respectively, and $\mu_{{a,\mathrm{EGFP}}_{\mathrm{ex}}}$ and $\mu_{{a,\;\mathrm{EGFP}}_{\mathrm{em}}}$ are the corresponding absorption coefficients. The emission factor has a negative sign because it does not contribute to absorption but is a source of outgoing (fluorescent) light.

The underlying absorption spectra for melanin, hemoglobin, and bilirubin were obtained from a database compiled by Jacques and Prahl,^37^ also published by Jacques.^14^ The absorption spectra for cytochrome C oxidase were obtained from Mason et al.,^38^ and the EGFP fluorescence emission and excitation spectra were obtained from FPbase.^39^ The hyperspectral images were fitted with a GPU-accelerated Levenberg–Marquardt algorithm to extract tissue model parameters and uncertainties (fitting errors). In the present study, all 11 (or 13 in the case with included EGFP) tissue model parameters were fitted to determine the total effect of renormalization and EGFP fluorescence inclusion on the fitting performance. Fitting was performed on a computer with an Nvidia Titan Xp graphics card with 12 GB RAM, an AMD Ryzen 7 1700X processor, and 16 GB RAM.

### 2.6 Glass Coverslips

We acquired the reflectance properties of various glass coverslips from different manufacturers/vendors to see whether the two GL100 glass coverslips utilized in DSWC models are representative of other coverslips. The details about glass coverslips are presented in Table 1. The coverslips ranged from 12 mm (in diameter), similar to GL100, to microscope cover glasses (from 18  mm×18  mm up to 24  mm×60  mm in size).

**Table 1:** Glass coverslips used in the study.

Glass coverslip	Size/diameter	Manufacturer/vendor
GLC1	Φ 12 mm	APJ Trading
GLC2	24 mm × 60 mm	ibidi
GLC3	18 mm × 18 mm	ZEISS
GLC4	24 mm × 24 mm	BRAND
GLC5	24 mm × 50 mm	Knittel Glass
GLC6	24 mm × 40 mm	VWR
GLC7	Φ 11 mm	Thermo Scientific
GLC8	18 mm × 18 mm	VWR

### 2.7 Tissue Phantoms

Furthermore, we utilized different tissue-mimicking phantoms to acquire hyperspectral images of the phantoms with and without the GLC1 glass coverslip placed on top of the phantoms. The phantoms were manufactured from resin (Clear Resin v4, Formlabs, Somerville, Massachusetts, United States) with added titanium dioxide (248576, Sigma-Aldrich, St. Louis, Missouri, United States) as scatterers and black pigment (Polycraft Silicone Pigment, MB Fibreglass, Newtownabbey, United Kingdom) as the absorber in different ratios to mimic various biological tissues. The phantoms were curated to speed up polymerization. The preparation of similar silicon-based phantoms was previously detailed by Rogelj et al.^33^

### 2.8 Human Forearm and Numerical Simulations

We performed an additional experiment in which two hyperspectral images of a single human forearm were acquired with and without a glass coverslip placed on top of the skin to collect a sample of data from which we could directly assess the effect of hyperspectral image renormalization. The approval of all ethical and experimental procedures and protocols in humans was granted by the Commission of the Republic of Slovenia for Medical Ethics (permission no. 0120-352/2022/3).

Moreover, we performed numerical simulations with the forward adding–doubling algorithm by assuming tissue parameters in Eq. (4) from the literature to create a generic tumor tissue with typical skin optical properties, including EGFP fluorescence.^35^ We fitted the acquired hyperspectral images of human forearms and generic simulated spectra using the IAD algorithm and assessed its performance in different scenarios.

### 2.9 Statistical Testing

To assess the relevance of the hyperspectral image renormalization and EGFP fluorescence modeling, we utilized the Mann–Whitney U-test.^40^ It is a non-parametric test that compares two independent groups to determine if there is a statistically significant difference between their distributions. Unlike parametric tests, it does not assume a normal distribution of the data, making it suitable for data that do not meet normality assumptions. It ranks combined data and evaluates whether the ranks between groups significantly differ. The U value reflects the number of times a value from one group is ranked higher than a value from another group. A significant p-value indicates a difference in distributions between the groups, providing robust evidence of a statistical difference.

Statistical testing was conducted using the *ranksum* function in MATLAB R2022b (Mathworks). The tested data consisted of tissue parameters extracted from either renormalized or non-renormalized hyperspectral images using the IAD algorithm based on either the 11-parameter model or the upgraded 13-parameter model. The specific details about the data are reported together with the results.

## 3 Results

Sections 3.1 and 3.2 present the results on tissue phantoms to identify the motivation and need for hyperspectral image renormalization. Section 3.3 supports the results with a simple human forearm experiment, and the motivation for EGFP fluorescence modeling is established based on numerical simulations. Finally, in Sec. 3.4, the results for the 4T1 tumor models in which biological variability plays a crucial role are presented, and the benefits of the two proposed methodological procedures are highlighted.

### 3.1 Glass Coverslips

**Fig. 3:**
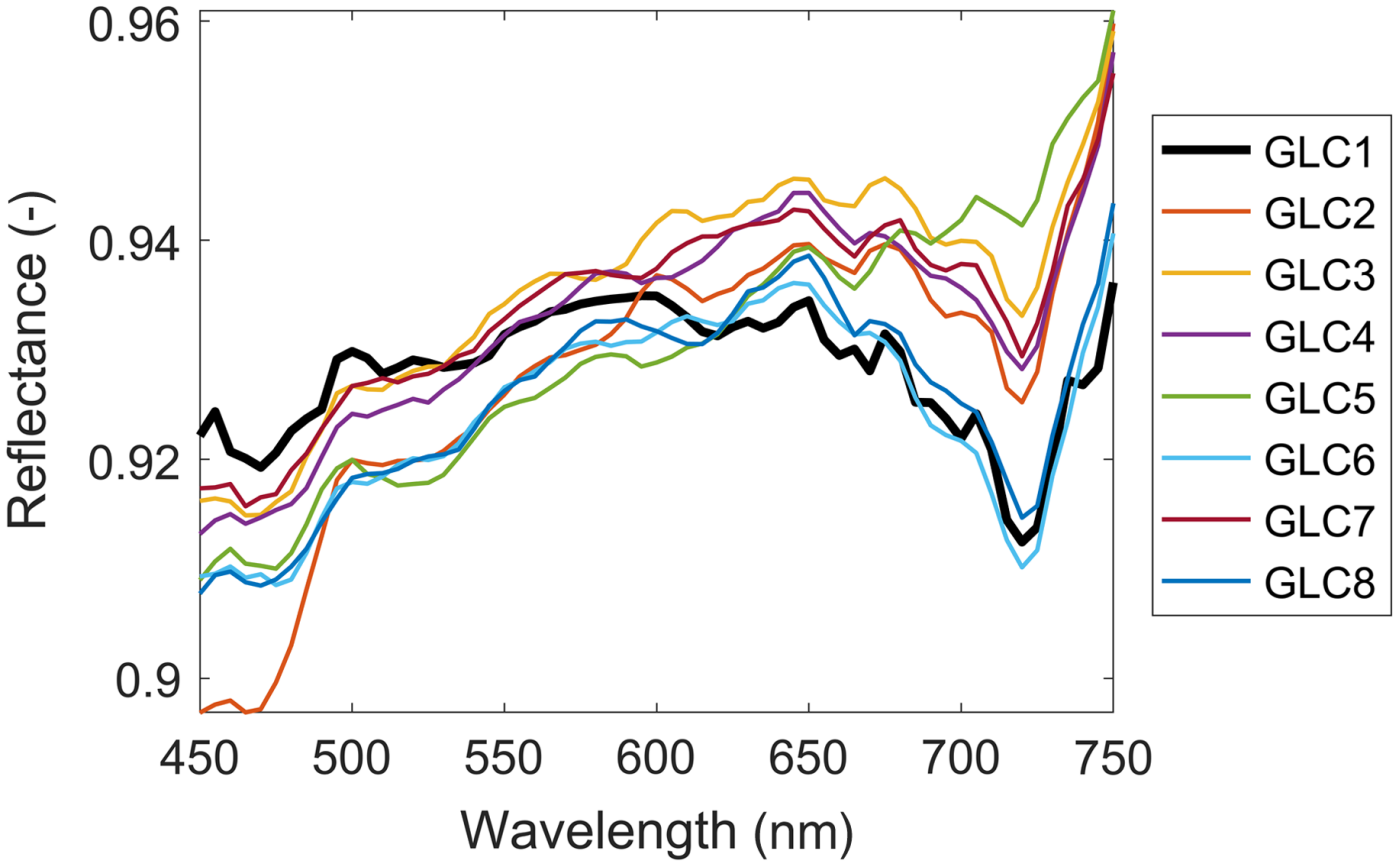
Average reflectance properties of various glass coverslips from different vendors placed on top of the white reflectance standard. GL100 coverslip utilized in DSWCs is presented as GLC1.

Figure 3 shows the average reflectance spectra of eight different glass coverslips placed on top of the white reflectance standard. We observed that values for all coverslips were between 0.9 and 0.96 in the spectral range of 450 to 750 nm and that the spectral properties of all glass coverslips resembled the properties of GL100 (named GLC1 in Fig. 3). The variability between coverslips was below 5%, from which we concluded that GL100 used in DSWCs is representative of other glass coverslips in terms of spectral properties.

### 3.2 Tissue Phantoms

**Fig. 4:**
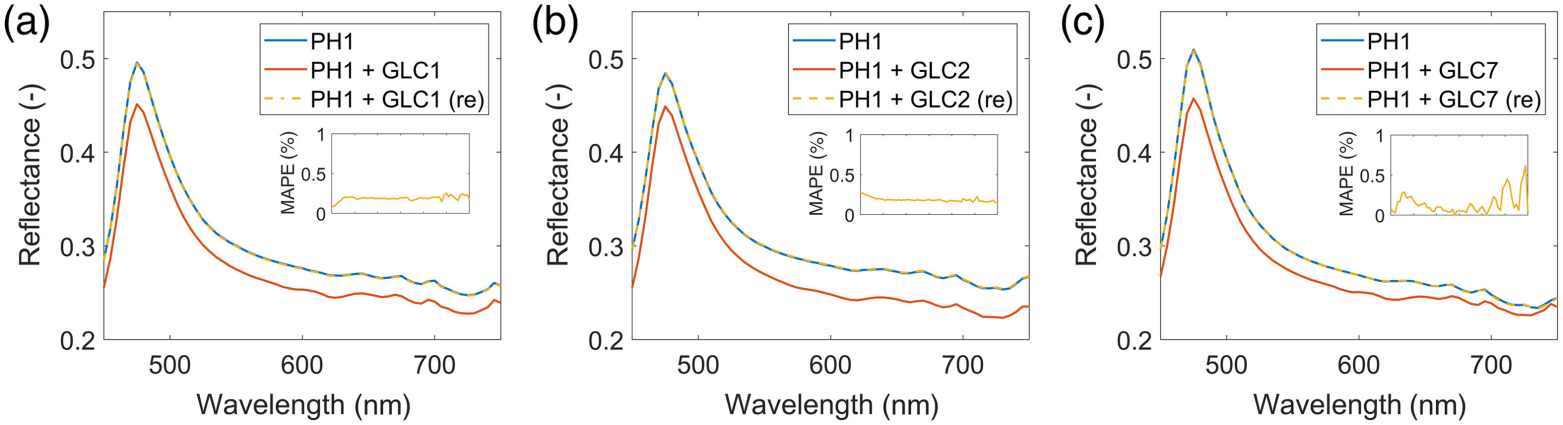
Average reflectance spectra of a PH1 tissue phantom without a glass coverslip (in blue) and with a glass coverslip placed on top of the PH1 phantom before renormalization (in orange) and after renormalization (in yellow). The results are shown for three selected coverslips: (a) GLC1 (GL100), (b) GLC2, and (c) GLC7. Insets show the MAPEs of the renormalized spectra of phantoms with glass coverslips (in yellow) with respect to the original spectra of phantoms without glass coverslips (in blue).

**Fig. 5:**
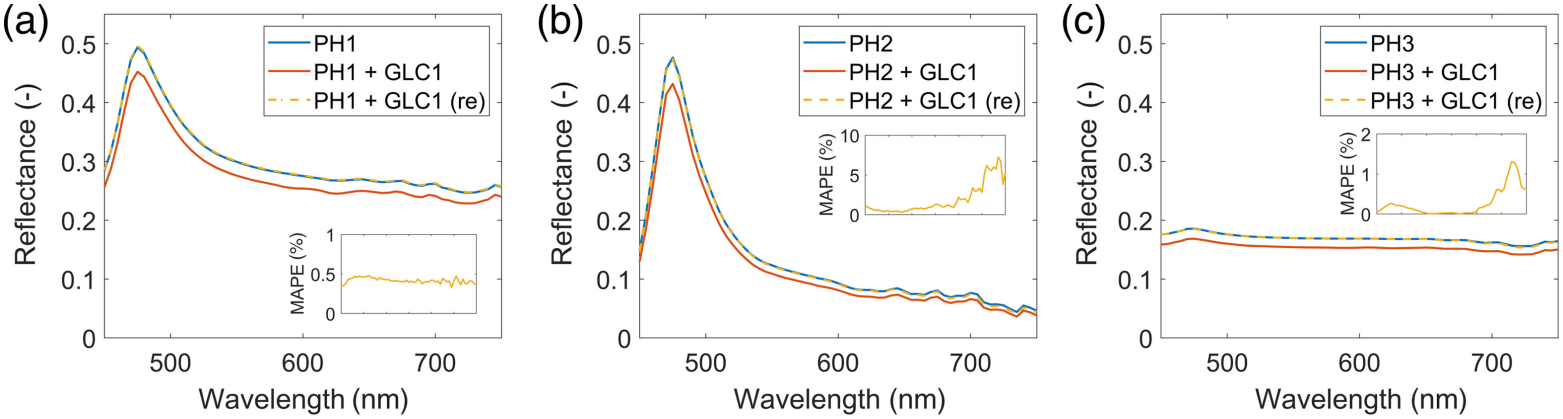
Average reflectance spectra of a tissue phantom without a glass coverslip (in blue) and with a glass coverslip placed on top of the phantom before renormalization (in orange) and after renormalization (in yellow). The results are shown for three selected tissue phantoms: (a) PH1, (b) PH2, and (c) PH3. All phantoms were covered with the GLC1 (GL100) glass coverslip. Insets show the MAPEs of the renormalized spectra of phantoms with glass coverslips (in yellow) with respect to the original spectra of phantoms without glass coverslips (in blue).

Figure 4 shows the average reflectance spectra of a PH1 tissue phantom with (in orange) and without (in blue) glass coverslips placed on top of the phantom. The measurement was repeated for three different glass coverslips: (a) GLC1 (GL100) utilized in DSWCs, (b) GLC2, and (c) GLC7. Moreover, Fig. 5 showcases the average reflectance spectra of three different tissue phantoms: (a) PH1 (resin with more scatterers), (b) PH3 (resin with less scatterers), and (c) PH3 (resin with absorber), with (in orange) and without (in blue) the GLC1 (GL100) glass coverslip placed on top of the phantom. In both figures, we noticed that the spectra of phantoms with coverslips after spectral renormalization (in yellow) closely matched the measured spectra of the phantoms without the coverslips (in blue). As the mean absolute percentage errors (MAPEs) of spectra reduced from ∼10% to below 2% in most cases, we concluded that spectral renormalization compensated for the light loss in the glass coverslips.

### 3.3 Human Forearm and Numerical Simulations

**Fig. 6:**
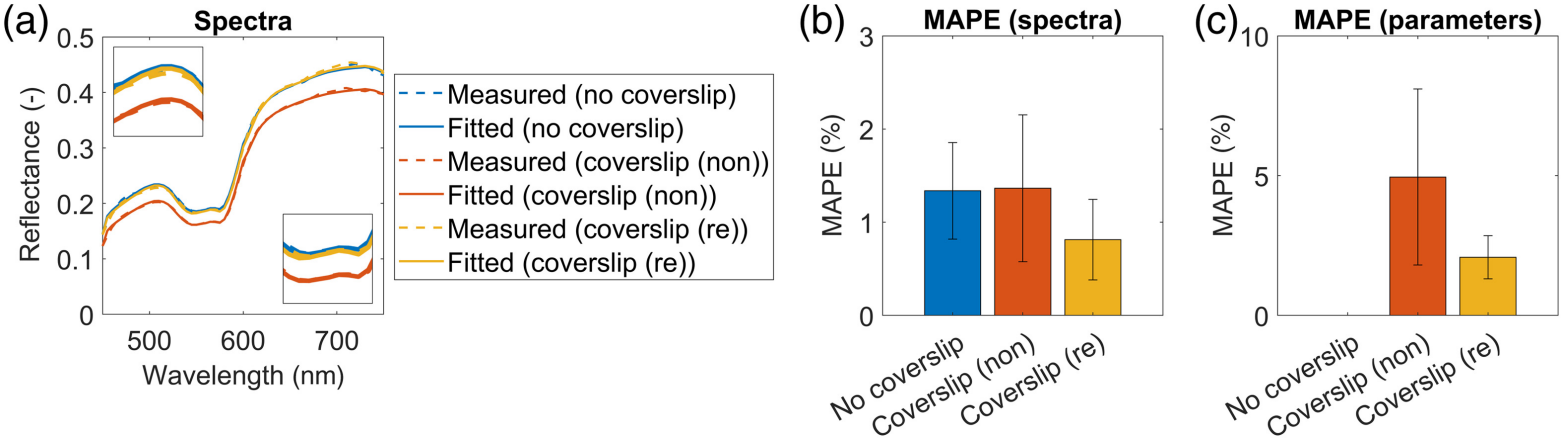
(a) Average measured (dashed curves) and fitted (solid curves) reflectance skin spectra of the human forearm with no coverslip placed on the skin (in blue) and with a coverslip placed on the skin before renormalization (in orange) and after renormalization (in yellow). The original 11-parameter model was used for IAD fitting in all cases. Insets show the 470- to 520- and 530- to 600-nm spectral ranges. (b) MAPE of fitted and measured reflectance skin spectra in the 450- to 750-nm spectral range. (c) MAPE of parameters extracted by the IAD algorithm. The results for parameters are shown just for the hyperspectral images of the forearm with the coverslips because the no coverslip parameters were considered ground truth. Error bars present the standard deviations of spectra and parameters.

Similarly, in the human forearm imaging experiment, we observed that the average spectrum of the hyperspectral image with the coverslip agreed more closely with the no coverslip image (in blue) after renormalization (in yellow) than before spectral renormalization (in orange), as seen in Fig. 6(a). This was also reflected in a lower MAPE for both spectra [Fig. 6(b), MAPE reduced by 0.55%] and parameters extracted with the IAD algorithm [Fig. 6(c), MAPE reduced by almost 3%]. The MAPE of parameters was calculated as the mean values of percentage errors for all fitted parameters. A statistically significant difference (p=0.021) between the parameters before and after renormalization was determined using the Mann–Whitney U-test. Statistical testing was performed on the pixel level, i.e., a dataset of all tissue parameters extracted with the IAD algorithm from the hyperspectral image of a human forearm before and after renormalization. Those results support our claim that hyperspectral image renormalization improves IAD performance, especially precision and robustness.

**Fig. 7:**
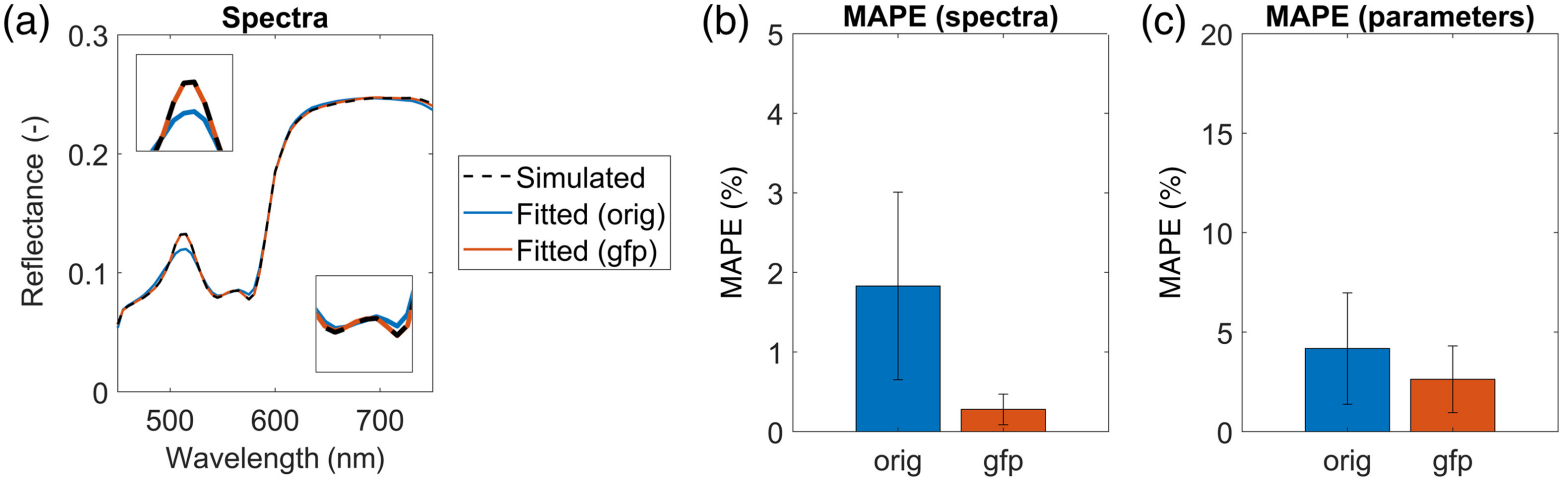
(a) Average simulated (dashed curve) and fitted (solid curves) reflectance skin spectra of a 4T1 tumor. The fitted spectra shown are for the original 11-parameter model (in blue) and the upgraded 13-parameter model (in orange) incorporating EGFP fluorescence. Insets show the 470- to 520- and 530- to 600-nm spectral ranges. (b) MAPE of fitted and simulated reflectance skin spectra in the 450- to 750-nm spectral range for both models. (c) MAPE of parameters extracted by the IAD algorithm using the original and upgraded models. Error bars present the standard deviations of spectra and parameters.

Similarly, for the numerical simulations of a sample 4T1 tumor reflectance spectrum [Fig. 7(a), dashed curve], we noticed that the agreement between the spectrum fitted with the 13-parameter model (gfp, in orange) and the simulated spectrum (black dashed curve) improved compared with the one fitted with the original 11-parameter model (orig, in blue). This is also evident in Fig. 7(b), showing the MAPEs of fitted spectra, which were reduced by ∼1.5%. Notably, the extracted parameters were also more precise for the 13-parameter model. As seen in Fig. 7(c), parameter MAPE was reduced by ∼1.5%, and we found a statistically significant difference (p=0.046) between the parameter values using the Mann–Whitney test. Statistical testing was performed on a dataset of all tissue parameters extracted with the IAD algorithm from the numerically simulated 4T1 tumor reflectance spectrum using the 11-parameter or 13-parameter model. Although the errors are substantial for the 11-parameter model (orig, in blue), the errors were reduced considerably for the 13-parameter model (gfp, in orange). Those simulations confirm our previous statements that considering EGFP in the murine skin model improves the performance of the IAD algorithm in tumors expressing EGFP.

### 3.4 Murine Tumor Models

This section describes the results obtained for hyperspectral image fitting using the IAD algorithm for non-renormalized and renormalized [Eq. (2)] hyperspectral images of 4T1 tumors grown in DSWCs. In both cases, the results are presented for two different skin models: the original 11-parameter model [Eq. (3)] and the upgraded 13-parameter model incorporating EGFP fluorescence [Eq. (4)].

#### 3.4.1 Spectral features

**Fig. 8:**
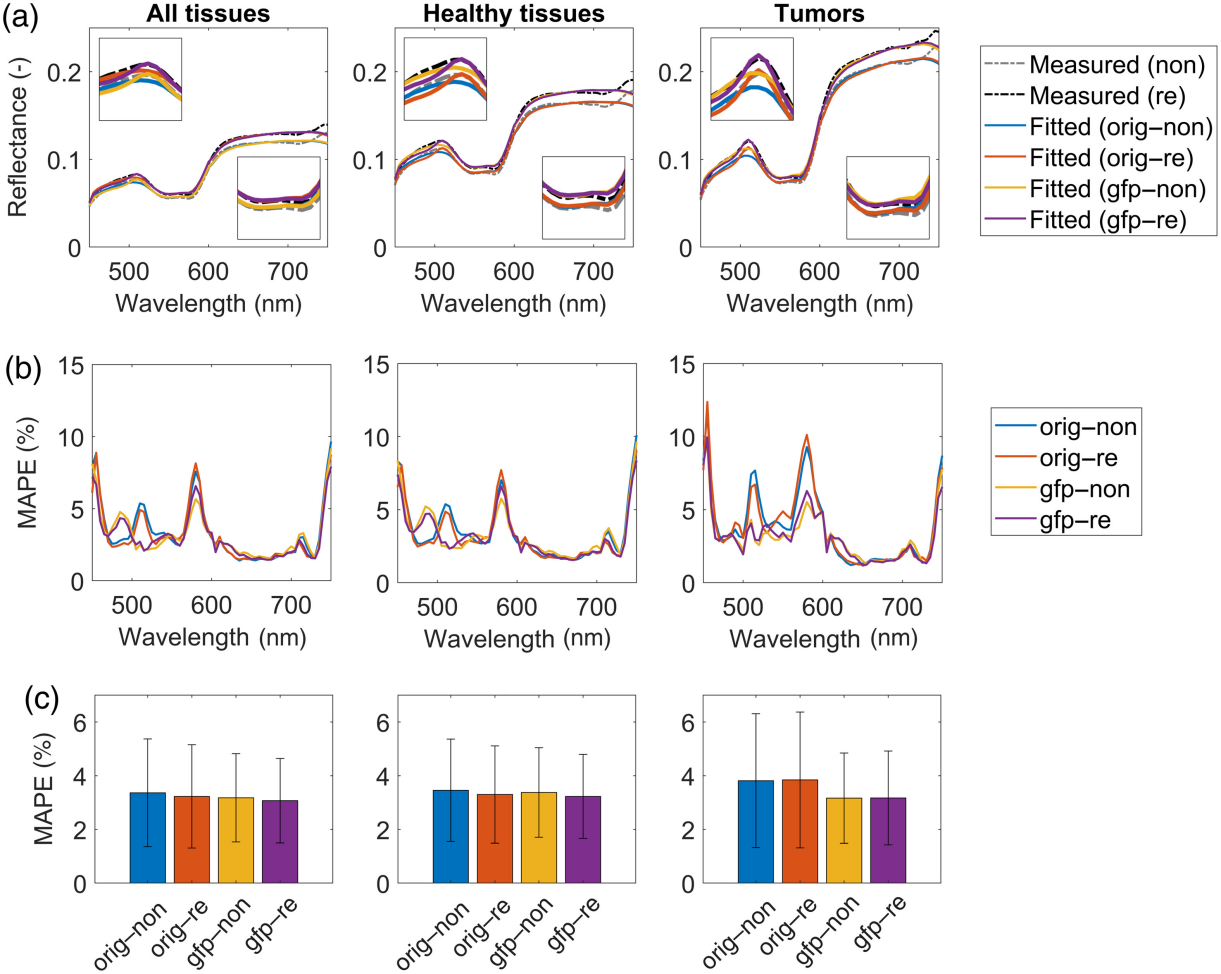
(a) Average measured and fitted reflectance skin spectra before and after renormalization for all tissues (left column), healthy tissues (middle column), and tumors (right column). The fitted spectra are shown for the original 11-parameter model (in blue for non-renormalized and orange for renormalized spectra) and the upgraded 13-parameter model incorporating EGFP (in yellow for non-renormalized and purple for renormalized spectra). Insets show the 470- to 520- and 530- to 600-nm spectral ranges where EGFP and hemoglobin properties are most pronounced, respectively. (b) MAPE of fitted and measured reflectance skin spectra in the 450- to 750-nm spectral range. (c) Overall MAPEs for different scenarios and the corresponding standard deviations presented as error bars. Legend: non, non-renormalized; re, renormalized; orig, original 11-parameter skin model; gfp, upgraded 13-parameter skin model incorporating EGFP.

To begin with, Fig. 8(a) shows the average measured reflectance spectra in the 450 to 750 nm spectral range before (non; grey dashed curves) and after renormalization (re; black dashed curves) for all tissues (left) and separately for healthy tissues (middle) and tumors (right). The average spectra were calculated by averaging the spectra of all seven subjects in the study acquired over the 14-day period, starting on the day of tumor cell inoculation. The healthy tissues comprised all tissues except for the tumors and the additional 1-mm tumor margins surrounding the tumors. Also shown in Fig. 8(a) are the fitted spectra for different scenarios: (1) original model, non-renormalized images (orig–non; in blue); (2) original model, renormalized images (orig–re; in orange); (3) upgraded model with EGFP, non-renormalized images (gfp–non; in yellow); and (4) upgraded model with EGFP, renormalized images (gfp–re; in purple). The spectra were averaged across all subjects and time points to capture an overall effect of the two corrections proposed in this work.

The average IAD-fitted spectra generally agreed well with the average measured spectra [see Fig. 8(a)]. The most considerable discrepancies between the measured and fitted spectra were in the 550- to 600-nm spectral interval, below 465 nm, and above 730 nm, as seen in Fig. 8(b), which shows MAPEs of the fitted spectra. The former could be attributed to higher noise due to the specific HSI system illumination. The discrepancies were also notable for tumors at around 510 nm (EGFP fluorescence emission). The overall MAPE for different scenarios is presented in Fig. 8(c). Notably, the MAPE values across all wavelengths were below 4%. Interestingly, the highest MAPEs and MAPE uncertainties were for the orig–non scenario, followed by orig–re, gfp–non, and gfp–re [see Fig. 8(c), left]. This indicates that both renormalization and the addition of EGFP fluorescence improved the matching of fitted and measured reflectance skin spectra in mice. For tumors, however, spectral renormalization decreased the fitting performance minorly. On the other hand, adding EGFP significantly improved the fitting of both non-renormalized and renormalized hyperspectral images. Although statistical testing was performed with the Mann–Whitney U-test on the pixel level, i.e., a dataset of all tissue parameters from all subjects on all days, no statistical significance was found due to the high biological variability between the subjects and for each subject itself due to tumor progression.

**Fig. 9:**
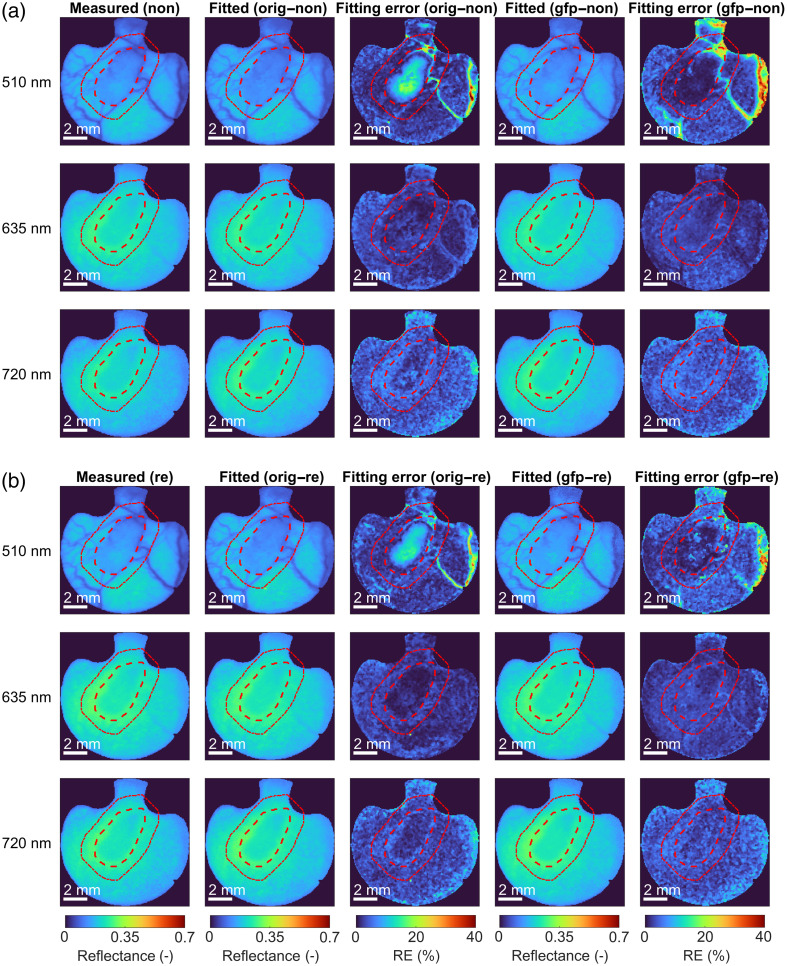
Measured and fitted spectral bands and the corresponding fitting errors for 510-, 635-, and 720-nm spectral bands for (a) non-renormalized and (b) renormalized hyperspectral images of subject 4 with a 4T1 tumor on day 10. The spectral bands shown here were fitted using the original 11-parameter and the upgraded 13-parameter murine skin model. The corresponding errors presented are REs. The tumor is delineated with a red dashed line, and the additional 1-mm tumor margin is with a red dotted line.

Furthermore, Fig. 9 shows the selected spectral bands (i.e., 510, 635, and 720 nm) obtained from the hyperspectral image of subject 4 on day 10 with a 4T1 tumor (column 1), the IAD-fitted spectral bands (columns 2 and 4), and the corresponding fitting errors (columns 3 and 5) presented as relative errors (REs). Figure 9(a) presents the results for non-renormalized spectral bands, and Fig. 4(b) presents the results for renormalized spectral bands. The tumor is delineated with a red dashed line, and the additional 1-mm tumor margin around the tumor is delineated with a red dotted line. Subject 4, showcased in Fig. 9 and subsequent figures, was selected as a representative example of the entire cohort, having a well-developed tumor vasculature and vasculature supplying the tumor. As such, it provides insight into the heterogeneous tumor microenvironment.

In all scenarios, the fitted spectral bands appeared very similar to measured spectral bands, and the fitting errors were generally below 4%, as already seen in Fig. 8(c). However, the spatial distribution of errors identified the regions of high fitting uncertainty, predominantly in the blood vessels due to low reflectance values as a result of high absorption in the blood. There was also a high error in the tumor region for the two scenarios in which the original model that did not consider EGFP fluorescence is used [row 1, column 3 in Figs. 9(a) and 9(b)]. However, with the addition of EGFP fluorescence in the upgraded model, the RE of IAD-fitting reduced substantially [row 1, column 5 in Figs. 9(a) and 9(b)].

#### 3.4.2 Tissue parameters

**Fig. 10:**
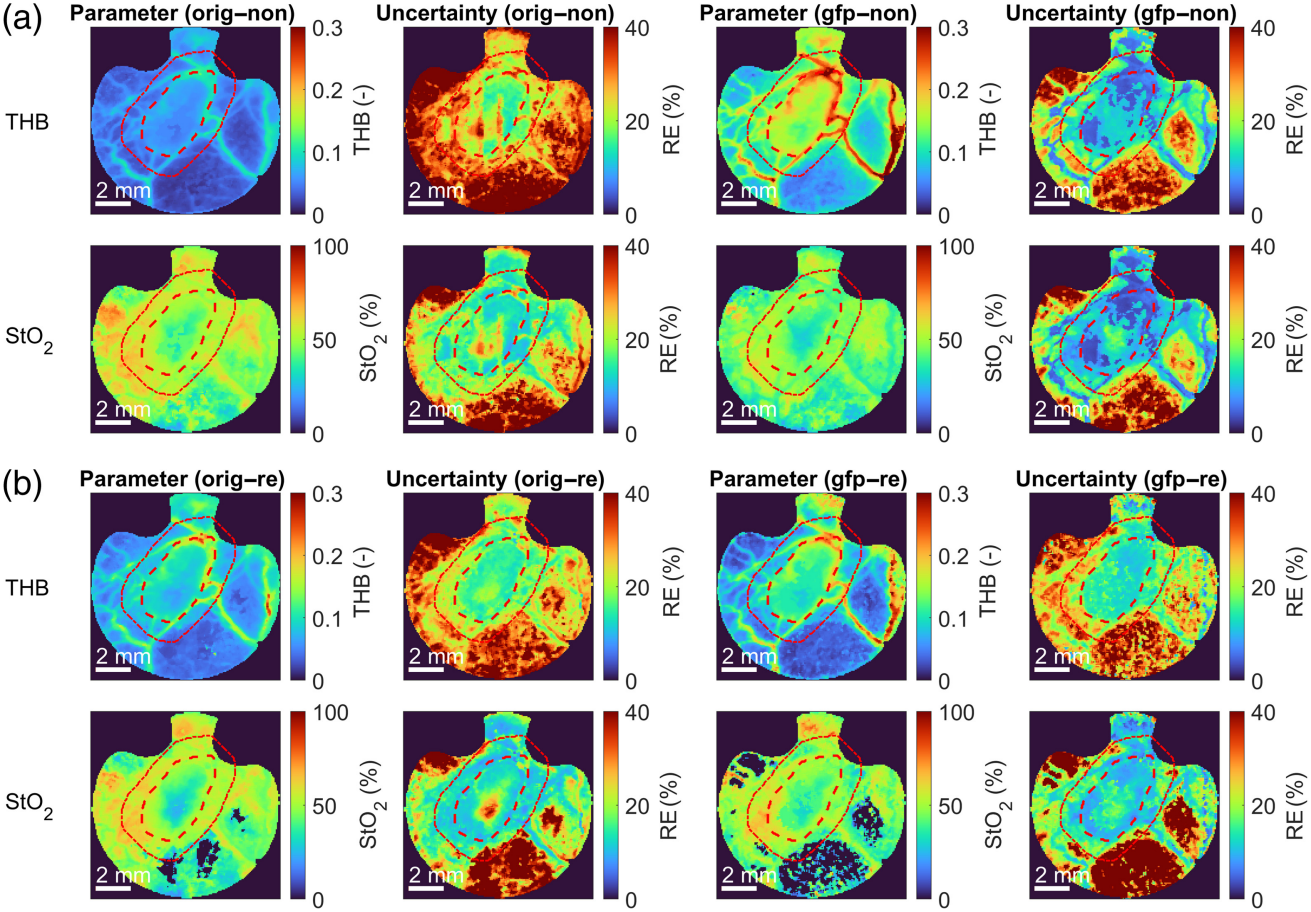
Color maps of THB and ${\mathrm{StO}}_{2}$ parameters and the corresponding parameter uncertainties extracted from (a) non-renormalized and (b) renormalized hyperspectral images of subject 4 with a 4T1 tumor on day 10 using the original 11-parameter and the upgraded 13-parameter murine skin model. The corresponding parameter uncertainties are presented as REs. The tumor is delineated with a red dashed line, and the additional 1-mm tumor margin is with a red dotted line.

Because the renormalization of hyperspectral images and the selection of the skin model affected the fitting performance of the IAD algorithm, this has also manifested in the tissue parameters extracted from the hyperspectral images. Figures 10(a) and 10(b) present the color maps of the total hemoglobin (THB) and ${\mathrm{StO}}_{2}$ and the corresponding parameter uncertainties (fitting errors) determined from the non-renormalized and renormalized hyperspectral images for subject 4 with a 4T1 tumor acquired on day 10, respectively. Although the color maps appeared similar at first sight, some variations existed for different scenarios. For example, renormalization and EGFP addition improved the image contrast for this particular subject. Also, the EGFP addition reduced the RE, especially in the tumor region.

**Fig. 11:**
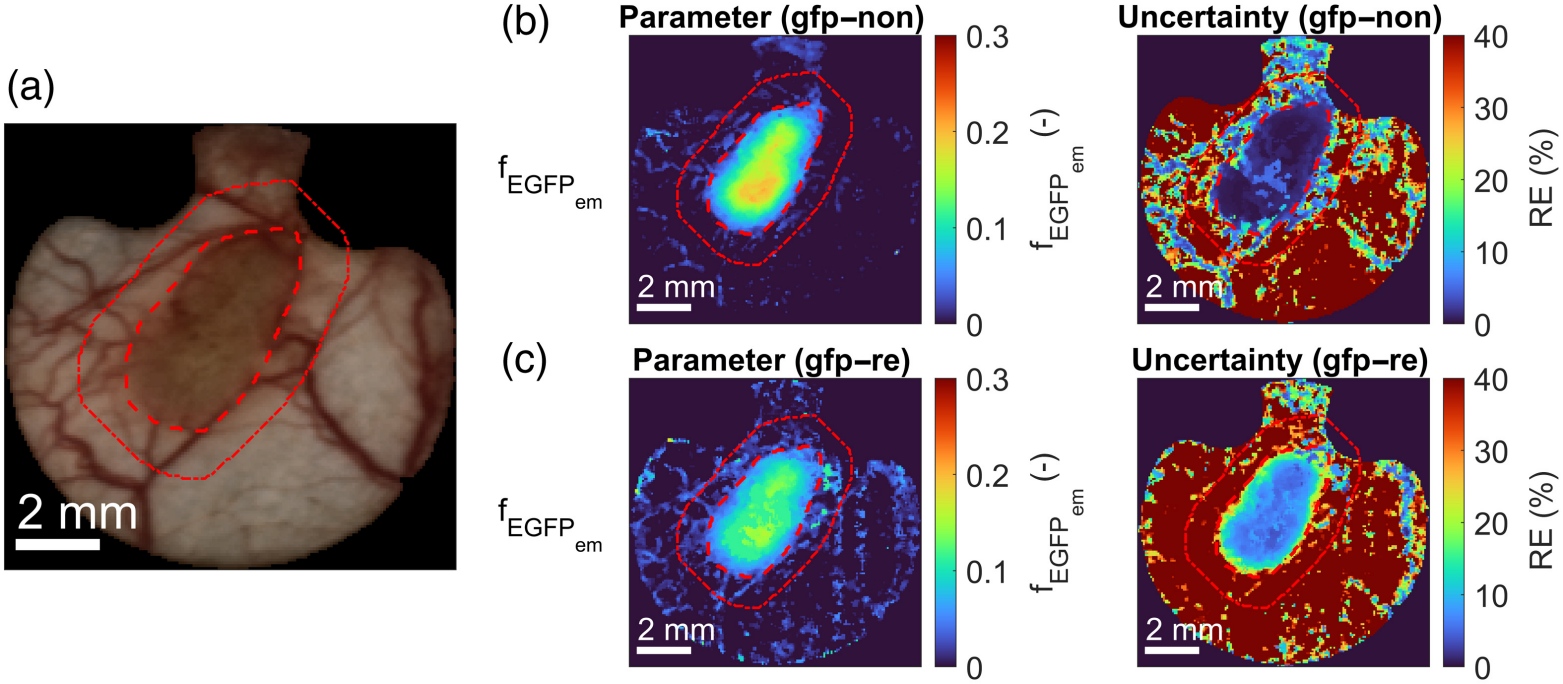
(a) RGB image extracted from the hyperspectral image images of subject 4 with a 4T1 tumor on day 10. Color maps of the EGFP fluorescence emission intensity ($f_{{\mathrm{EGFP}}_{\mathrm{em}}}$) parameter and the corresponding parameter uncertainties extracted from (b) non-renormalized and (c) renormalized hyperspectral images of a subject with a 4T1 tumor using the upgraded 13-parameter murine skin model that considered EGFP fluorescence. The corresponding parameter uncertainties are presented as REs. The tumor is delineated with a red dashed line, and the additional 1-mm tumor margin is with a red dotted line.

Similarly, shown in Figs. 11(b) and 11(c) are the color maps of EGFP fluorescence emission intensity ($f_{{\mathrm{EGFP}}_{\mathrm{em}}}$) for the upgraded model before and after renormalization alongside the RGB image. Note that the original 11-parameter model did not consider the $f_{{\mathrm{EGFP}}_{\mathrm{em}}}$ parameter and is thus not shown. Most notably, the fitting errors in the tumor area were much higher for the renormalized image of subject 4 on day 10 than for the non-renormalized image. Also, the fitting errors in the healthy tissues were very high due to the low $f_{{\mathrm{EGFP}}_{\mathrm{em}}}$ parameter values. We can also see that 4T1 tumor segmentation could be performed based on the $f_{{\mathrm{EGFP}}_{\mathrm{em}}}$ parameter, as the $f_{{\mathrm{EGFP}}_{\mathrm{em}}}$ distribution agrees well with the manual segmentations (red dashed lines). A trained expert performed the manual segmentation of tumors in Fiji^41^ software based on the RGB images of tumors extracted from hyperspectral images, as seen in Fig. 11(a). An additional 1-mm tumor margin with a possible tumor spread was also delineated.

**Fig. 12:**
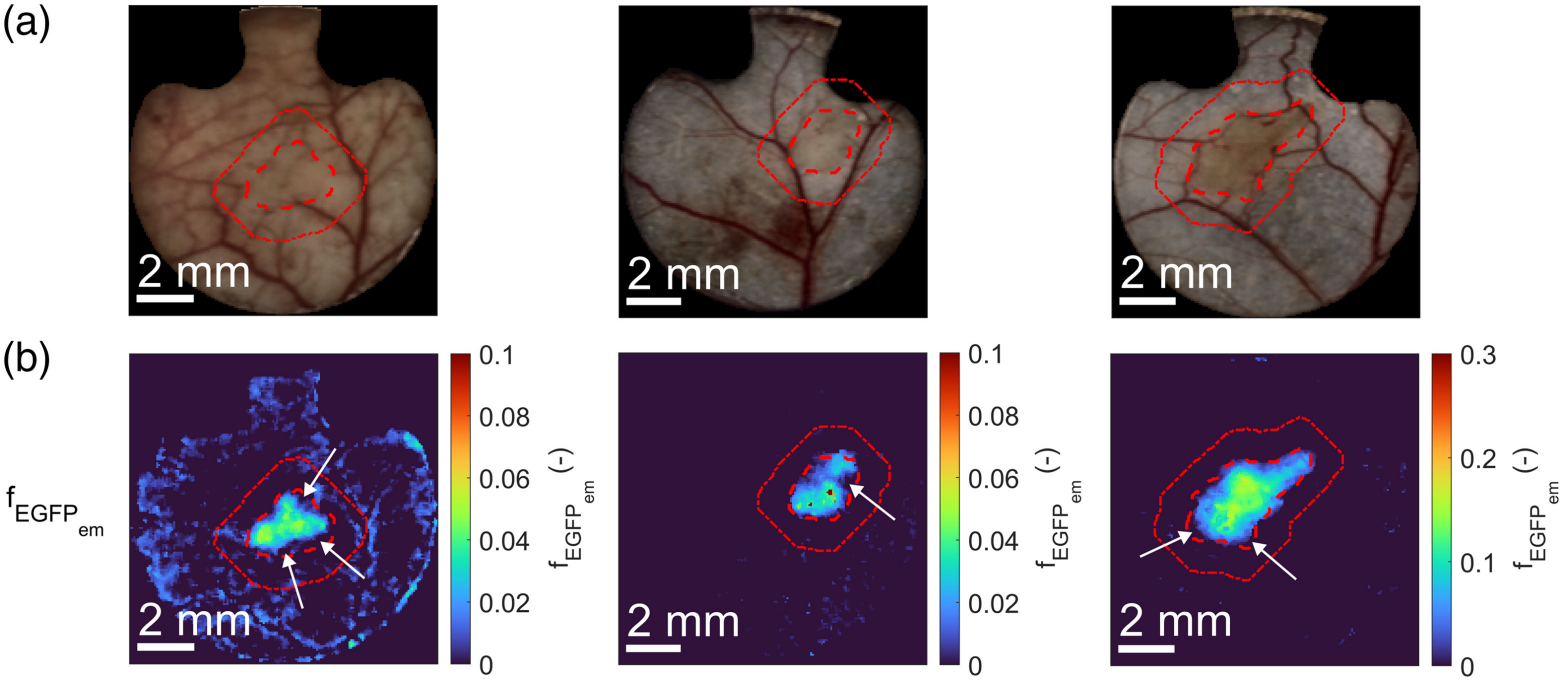
(a) RGB images and (b) color maps of EGFP fluorescence emission intensity ($f_{{\mathrm{EGFP}}_{\mathrm{em}}}$) parameter extracted from renormalized hyperspectral images using the upgraded 13-parameter murine skin model that considered EGFP fluorescence. The first and second columns are subjects 3 and 7 on day 3, respectively, and the last column is subject 5 on day 5. The tumor is delineated with a red dashed line, and the additional 1-mm tumor margin is with a red dotted line. White arrows point at regions where manual segmentations could be improved with the information about $f_{{\mathrm{EGFP}}_{\mathrm{em}}}$ distribution.

Specifically, the $f_{{\mathrm{EGFP}}_{\mathrm{em}}}$ parameter distribution could improve the manual tumor segmentations at early stages or enable automatic segmentation, as seen in Fig. 12, which shows the RGB images and $f_{{\mathrm{EGFP}}_{\mathrm{em}}}$ color maps of subject 3 on day 3 (first column), subject 7 on day 3 (second column), and subject 5 on day 5 (last column). White arrows point at regions where a mismatch was found between the manual tumor segmentations and regions of high $f_{{\mathrm{EGFP}}_{\mathrm{em}}}$ intensity and where information about $f_{{\mathrm{EGFP}}_{\mathrm{em}}}$ distribution could, therefore, improve the manual segmentations. In most cases, the manual segmentations overestimated the extent of the tumor due to a conservative approach to delineating tumor margins.

**Fig. 13:**
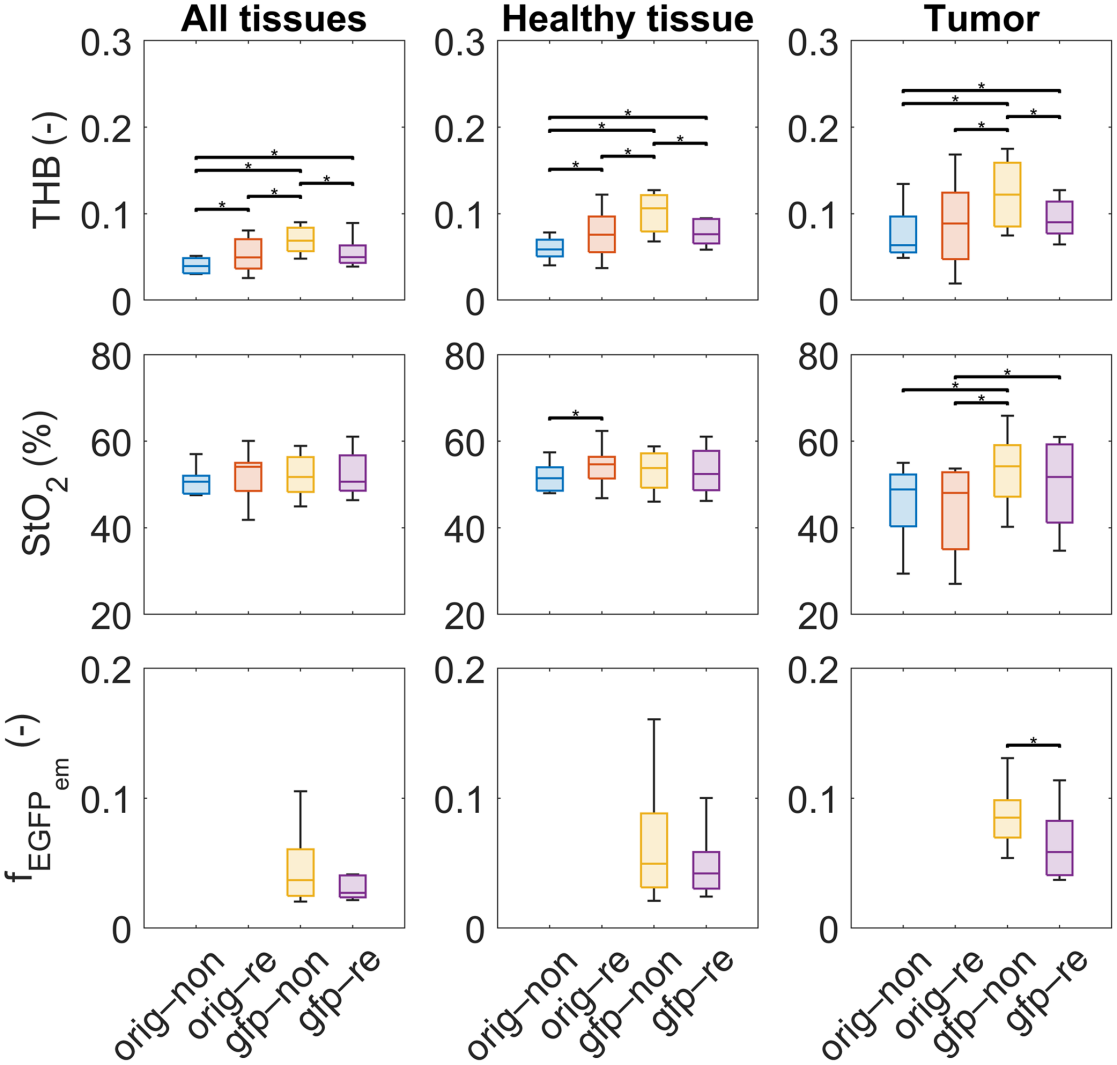
Average THB, ${\mathrm{StO}}_{2}$, and EGFP fluorescence emission intensity ($f_{{\mathrm{EGFP}}_{\mathrm{em}}}$) value images of subject 4 with a 4T1 tumor on day 10 for different scenarios. Error bars represent the standard deviations of parameters. Asterisk (*) shows pairs of parameter values for different scenarios, for which the Mann–Whitney U-test found a statistically significant difference (p<0.05). Legend: non, non-renormalized; re, renormalized; orig, original 11-parameter skin model; gfp, upgraded 13-parameter skin model incorporating EGFP.

Because we found no statistical significance in Fig. 8(c), where tissue parameters of all subjects on all days were compared, we focused on the parameters of a single subject on a single day to reduce inter- and intrasubject variability as much as possible. Figure 13 shows the box plots of estimated tissue parameters for all tissues (left), healthy tissues (middle), and tumors (right) for all four scenarios and a single subject (subject 4 on day 10) with a 4T1 tumor. Importantly, we observed that hyperspectral image renormalization and EGFP fluorescence modeling affected the values of tissue parameters estimated using the IAD algorithm. The most notable changes were observed for THB, for which the Mann–Whitney U-test confirmed the statistically significant differences (p<0.05) for multiple pairs of scenarios, as denoted by asterisks (*) in the graphs. Statistical testing was performed on the pixel level, i.e., a dataset of a specific tissue parameter (e.g., THB) extracted with the IAD algorithm from hyperspectral images using two scenarios (e.g., orig–non and orig–re), and all pairs of scenarios were tested. The alteration in the ${\mathrm{StO}}_{2}$ parameter was generally less pronounced than for THB, except for tumor tissue. Similar results were obtained for $f_{{\mathrm{EGFP}}_{\mathrm{em}}}$. Box plots of the $f_{{\mathrm{EGFP}}_{\mathrm{em}}}$ parameter for the original model are not shown as the parameter was not considered in the model.

**Fig. 14:**
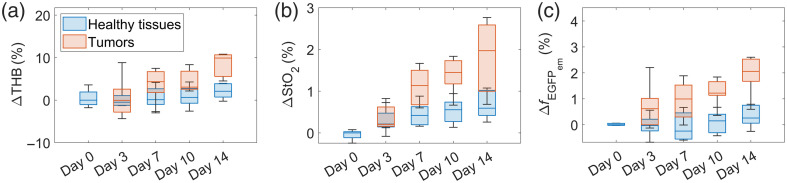
Changes in baseline tissue parameter values of (a) THB, (b) ${\mathrm{StO}}_{2}$, and (c) EGFP fluorescence emission intensity ($f_{{\mathrm{EGFP}}_{\mathrm{em}}}$) for healthy tissues (in blue) and tumors (in orange) for all subjects on days 0, 3, 7, 10, and 14.

Finally, we leveraged the gfp–re scenario to extract tissue properties of 4T1 tumors and surrounding healthy tissues over the course of 14 days. Figure 14 shows the changes in baseline values of (a) THB, (b) ${\mathrm{StO}}_{2}$, and (c) $f_{{\mathrm{EGFP}}_{\mathrm{em}}}$ for tumors and healthy tissues for all subjects included in the study at different time points. The changes are calculated so that the median value for healthy tissues on day 0 equals 0%. For all parameters, the longitudinal changes were more evident for tumors than for healthy tissues. For example, the most considerable changes from days 0 to 14 were observed for THB (9.9% in tumors and 2.1% in healthy tissues), and the median $\Delta f_{{\mathrm{EGFP}}_{\mathrm{em}}}$ on day 14 for tumors and healthy tissues was 2.1% and 0.3%, respectively. This suggests that the optical properties of tumors alter considerably as tumors grow and that the differentiation between tumors and healthy tissue is improved as the disease progresses, although the biological variability is high.

## 4 Discussion

This study highlights the often-overlooked problem with HSI (and optical imaging in general) of biological tissues monitored within DSWCs. Light impeding on the glass coverslip of a DSWC is partly specularly reflected off the glass surface, and the majority is transmitted through the glass and enters the biological tissue, undergoing absorption and scattering. In reflectance imaging mode, the light interacting with the tissue must exit and pass through the glass coverslip again before reaching the detector [Fig. 1(b)].

Many imaging systems, including our custom-built HSI system,^31^ use crossed polarizers to reduce specular reflection and compensate for the apparent increase in overall reflectance. However, to the best of our knowledge, no previous study utilizing HSI for DSWC imaging considered the loss of reflected light due to light passing through the glass coverslip in a DSWC.

In the experiments involving tissue-mimicking phantoms (Figs. 4 and 5), we showed that the spectral renormalization compensated for the light loss in the glass coverslips by improving the agreement between the measured spectra of tissue phantoms with and without coverslips, in which the former underwent spectral renormalization. By measuring the reflectance properties of various glass coverslips (Fig. 3) and showing that the variations are below 5%, we confirmed that the results could be generalized to coverslips from different vendors and for different applications.

With the two additional experiments involving human forearm imaging and numerical simulations, we supported our findings that hyperspectral image renormalization and EGFP fluorescence modeling improve the agreement of the spectra. Moreover, we demonstrated that the values of parameters before and after renormalization, as well as before and after the EGFP addition, were significantly different (p=0.021 and p=0.046, respectively). All in all, we showed that the spectral renormalization of hyperspectral images [see Eq. (2)] based on measured average reflectance spectra of glass coverslips [see Figs. 2(b) and 2(c)] improved the overall performance of the IAD algorithm for extracting tissue properties from hyperspectral images. We also confirmed that the hyperspectral image renormalization could be generalized to other optical imaging applications involving glass coverslips by measuring the reflectance properties of coverslips and applying renormalization, as described in Eq. (2). Therefore, the renormalization process represents an advancement in quantitative imaging as it considers and compensates for errors inherent in experimental procedures.

We then applied the hyperspectral image renormalization and EGFP modeling to the murine tumor models. Although the fitted spectra for all four presented scenarios generally agreed well with the measured spectra [see Fig. 8(a)], the MAPE and its uncertainty slightly reduced upon hyperspectral image renormalization, as seen in Fig. 8(c). This could be attributed to a marginal improvement in the signal-to-noise ratio (SNR) in measured spectra. For renormalized spectra, the SNR improved by as much as 2 dB (4% of the average value), and the SNR uncertainty almost halved in some cases. Those results align with our previous findings, showing that adding noise to reflectance spectra deteriorates the IAD algorithm performance.^35^ In the present study, hyperspectral image renormalization improved the SNR (reduced noise), leading to an enhanced fitting performance.

However, we noticed that the fitting error in the tumor region was significant, particularly in the spectral region between 480 and 520 nm, because the 4T1 tumor expressed a gene to produce EGFP. Although the EGFP excitation (absorption) was less evident, the fluorescence emission peaking at around 510 nm was highly pronounced in the reflectance spectra of the tumors [see Fig. 8(a)]. Therefore, we upgraded the original 11-parameter murine skin model to a 13-parameter model that also considered EGFP excitation and emission.

Doing this reduced the relative fitting error in the tumor region by almost 15%, as seen from 510-nm spectral bands in Figs. 9(a) and 9(b). This is because the fitted spectra matching with measured spectra improved significantly, as seen by comparing the yellow curves with the purple curves in Fig. 8(a) (right). The original model generally underestimated the reflectance around the 510-nm emission peak and slightly overestimated it in the 450- to 500-nm spectral region compared with the upgraded EGFP model. For all wavelengths, the MAPE of spectra decreased after introducing two additional EGFP-related parameters in the model. The most notable drop in MAPE was observed in tumors by around 0.7 percentage points from 3.81% (orig–non) to 3.17% (gfp–re), as seen in Fig. 8(c) (right), which is a 17% relative decrease in MAPE compared to the baseline value. Those results, in combination with simulations presented in Fig. 7, confirm that considering EGFP fluorescence in the murine skin model improved the overall performance of the IAD algorithm. However, the improvement was more notable in simulations (see Fig. 7) than in murine tumor models due to high biological variability, but it was also statistically significant in tumor tissue.

Most importantly, adding EGFP fluorescence in the model allowed us to localize the tumor based on EGFP emission, as seen in Fig. 11. Because the $f_{{\mathrm{EGFP}}_{\mathrm{em}}}$ parameter corresponds well with the manual segmentations (red dashed lines in Fig. 11), fluorescence emission could be used for 4T1 tumor segmentation. This could prove helpful in two cases: (a) it could aid in manual tumor segmentation, primarily to determine tumor margins in early days, as demonstrated in Fig. 12, where white arrows highlight the areas of mismatch between manual segmentations and high $f_{{\mathrm{EGFP}}_{\mathrm{em}}}$ intensity; (b) it could potentially enable the automatic segmentation of tumors with EGFP fluorescence based on the $f_{{\mathrm{EGFP}}_{\mathrm{em}}}$ parameter, which reduces the involvement of a trained professional and is thus considerably faster and cheaper.

Similarly, Sorg et al.^18^ and Palmer et al.^19^ localized 4T1 tumors with the help of fluorescence. As their tumor cells normally expressed red and green fluorescent proteins in hypoxic regions, they could also establish the extent of hypoxia in tumors. On the other hand, we could estimate hypoxia via the ${\mathrm{StO}}_{2}$ parameter representing tissue oxygenation.^36^ For example, we noted a lower ${\mathrm{StO}}_{2}$ value in the central region of the tumor for the subject in Figs. 10(a) and 10(b), possibly indicating hypoxia. We also observed increased THB values in the tumor (see Figs. 10(a) and 10(b)], which could be explained by a higher blood content due to demands related to tumor growth.^1^^,^^2^

As already mentioned, the performance of the fitting algorithm due to spectral renormalization and EGFP addition is also reflected in the values of tissue properties estimated from the hyperspectral images, as can be seen in Figs. 10 and 11. For example, the contrast between the tumor and the surrounding tissue improved for THB and ${\mathrm{StO}}_{2}$ in Figs. 10(a) and 10(b) in both scenarios. The numerical values presented for subject 4 on day 10 in Fig. 13 confirmed statistically significant differences (p<0.05) between some parameter distributions for different scenarios, especially for THB. However, the differences were less pronounced due to high tissue heterogeneity than in the human forearm experiment (Fig. 6) or the numerical simulations (Fig. 7).

Ultimately, we leveraged spectral renormalization and EGFP fluorescence modeling by evaluating the changes in baseline tissue parameter values of 4T1 tumors and surrounding healthy tissues over the course of 14 days, as seen in Fig. 14. We observed more pronounced changes in tumors than in healthy tissues, suggesting that optical properties of tumors change significantly as they grow and progress. Our results indicate that HSI could help detect tumors as early as possible based on the changes in blood content and other tumor-specific tissue parameters, such as EGFP fluorescence emission intensity. However, the biological variability observed is high, as seen in Fig. 14, and additional studies are needed to confirm this claim. As a result of high biological variability between different subjects and for each subject itself due to tumor progression, we also found no statistically significant differences in Fig. 8(c), where the Mann–Whitney U-test was performed on a dataset of all tissue parameters from all subjects on all days.

Nevertheless, as a sanity check, we attempted to replicate some previously reported results from the literature. For instance, the mean values of the epidermis and dermis thickness extracted with the IAD algorithm for all mice were 7.5±3.3 and 184.2±46.7  μm, respectively, reproducing results for Balb/c mice by Sabino et al.^42^

There are certain limitations to this study. We assumed that the light must pass through the coverslip twice in reflectance mode and then used the average spectrum of two glass coverslips [Fig. 2(b)] for renormalization. However, fluorescent light due to EGFP only passes through the coverslip once while impeding on the DSWC (for absorption) or after exiting the tissue (for emission). Due to our assumption, we slightly overestimated EGFP fluorescence intensity and normalized reflectance values in the spectral region below 520 nm.

Furthermore, as the tumors grew, they increasingly pressed on the glass coverslip from the bottom, creating a gap between the surrounding (healthy) tissue and the coverslip. The air filled the gap in most cases, but inflammation could also occur in tumors. However, we did not account for this possible refractive index mismatch due to the air pocket between the glass and the skin, which could affect the amount of light reflected on the detector. Moreover, we did not account for the differences in refractive indices among various tissue types. However, the refractive index mismatch is more significant at the air–glass interface than at the glass–biological tissue interface. Therefore, slight variations in tissue refractive indices have a minor impact on hyperspectral image renormalization.

In addition, the 13-parameter model is disadvantageous in some cases compared with the 11-parameter model, especially in the absence of EGFP fluorescence in healthy tissue. Because the model has two additional degrees of freedom, this could lead to less robust spectra fitting and tissue parameter extraction.

In the future, we propose improving a two-layer murine skin model to incorporate an additional layer of glass on top of the skin to refine the simulation of DSWCs for enhanced physiological relevance. Although our study focused on 4T1 tumors growing in DSWCs, the prospective utilization of this improved model extends to various therapeutic approaches in which DSWCs are utilized. Specifically, the improved murine skin model could be implemented for radiotherapy, electrochemotherapy, and reversible electroporation, given the significance of understanding changes in both tumors and surrounding healthy tissues. Incorporating such a model holds promise for advancing our understanding of tissue parameters before and after therapy to optimize treatment strategies and outcomes.

Moreover, we intend to study longitudinal changes in tumor characteristics in more detail in future investigations as understanding these dynamics could provide valuable insights into tumor biology.

## 5 Conclusion

This study introduced a novel approach to intravital monitoring of murine 4T1 mammary carcinomas grown in DSWCs using a custom-built HSI system. Specifically, the significance of renormalizing hyperspectral images to compensate for signal loss due to light interacting with glass coverslips was underscored, and a correction was proposed, providing a methodological advancement for precise tissue properties extraction using the IAD algorithm. In addition, the incorporation of EGFP fluorescence properties in the murine skin tissue model demonstrated enhancement in the performance of the IAD algorithm for hyperspectral image analysis and could be advantageous for the segmentation of tumors expressing EGFP. The proposed hyperspectral image renormalization process helps pave the way to more accurate and precise quantitative imaging as it considers and compensates for errors inherent in experimental procedures. Meanwhile, EGFP fluorescence modeling could enable automatic segmentation of tumors expressing EGFP and facilitate image processing and analysis. The improvement of HSI with proposed methodological refinements holds promise for advancing intravital imaging techniques in DSWC models, especially for tumor growth monitoring and early detection.

## Data Availability

The code, data, and materials that support the findings of this study are available from the corresponding author upon reasonable request.
